# Supplementary material for: Miocene restriction of the Pacific-North Atlantic throughflow strengthened Atlantic overturning circulation
Source: Nat Commun. 2019 Sep 6;10:4025. doi: 10.1038/s41467-019-12034-7 (PMC6731301; doi:10.1038/s41467-019-12034-7)
Supplement: Supplementary file 1 — Supplementary Information [file 41467_2019_12034_MOESM1_ESM.docx]

SUPPLEMENTARY INFORMATION

Miocene restriction of the Pacific-North Atlantic throughflow strengthened Atlantic overturning circulation

Valeriia Kirillova^a,^, Anne H. Osborne^a,*^, Tjördis Störling^a^, and Martin Frank^a^

^a^GEOMAR Helmholtz Centre for Ocean Research Kiel, Germany.

*Corresponding author: aosborne@geomar.de.

**SUPPLEMENTARY FIGURES**

Supplementary Figure 1 Comparison of seawater and coretop Nd isotope composition in the vicinity of Ocean Drilling Program Site 1006. Core top ε_Nd_ values (this study) together with water column ε_Nd_ and salinity profiles for Station 200-2^33^.

Supplementary Figure 2 Core top and seawater sampling sites. Location of core top samples taken from the vicinity of ODP Site 1006, as well as the position of the nearest available water column ε_Nd_ profile at Station 200-2^33^. Map produced using Ocean Data View^62^.

Supplementary Figure 3. Seawater and detrital fraction Nd isotope records. New seawater Nd isotope signatures extracted from uncleaned foraminifera and detrital fraction data for ODP Site 1006 from the Florida Straits and Site 1000 from the Caribbean Sea together with core top values of uncleaned foraminifera near ODP Site 1006 and published data for ODP Site 1000^34^.

**SUPPLEMENTARY DISCUSSION**

**ALTERNATIVE INTERPRETATIONS OF THE ND ISOTOPE RECORDS**

***Scenario 1: Balance between radiogenic Pacific waters and unradiogenic Atlantic waters (adopted as most likely interpretation in the manuscript)***

For the interpretation of the seawater Nd isotope record of Site 1006 we assume that the signal was always primarily a mixture between the Pacific outflow and Atlantic waters. When the Central American Seaway (CAS) was open, Pacific waters entered the Caribbean and, following modeling predictions, continued directly to the western North Atlantic via the Florida Strait, and/or continued eastward and were entrained into the Atlantic gyre. In either scenario, we attribute a more radiogenic ε_Nd_ signature at Site 1006 to a larger fraction of Pacific waters reaching the Site^6^. We attribute a less radiogenic ε_Nd_ signal at Site 1006 between 11.5 and 9.5 Ma to a cut-off from the Pacific supply and assume that seawater in the North Atlantic gyre without the Pacific contribution was always less radiogenic than Pacific intermediate waters. Although there are no other intermediate depth records available this assumption is justified given that all deeper records from the Atlantic have remained unchanged during the studied period of time (cf. ref. 7). The fact that Site 1000, 1006 and Blake records were indistinguishable between 8.5 and 8 Ma supports the scenario of a strong, direct throughflow between the Sites. Moreover, based on Nd and Pb isotope records for the Blake crust, Reynolds et al.^28^ argued for a strong Pacific signal still reaching the North Atlantic at 8.5 Ma and decreasing thereafter.

***Scenario 2: (Marked) Change in the Atlantic end-member***

The interpretation of intermediate depth ε_Nd_ records in the North Atlantic is strongly dependent on the consequences of changes of AMOC strength^29^. A stronger AMOC could potentially deliver more radiogenic waters of South Atlantic origin to the Florida straits, which could serve as an alternative explanation for more positive ε_Nd_ signatures.

Data obtained from ferromanganese crusts^64^ and sediments^65^ show that the Southern Ocean end-member remained on average at -10 to -7 ε_Nd_ over the past 14 Myr. Therefore, changes in this end-member cannot account for the highly radiogenic values seen in the Site 1006 record prior to 11.5 Ma and the Site 1000 record prior to 9.5 Ma without invoking a direct Pacific contribution via the CAS. The Caribbean volcanic island arcs are a potential source of radiogenic ε_Nd_ to recirculating Atlantic gyre waters but these islands are also present today and have no discernable impact on the ε_Nd_ composition of seawater at the water depth of our study^33^.

In summary, when the Site 1006 ε_Nd_ signatures were similar to those of the Site 1000 record, we can be confident that the radiogenic signal originated in the Pacific. When the Site 1006 ε_Nd_ record was less radiogenic than the Site 1000 record, this indicates that the export of Pacific waters to the western North Atlantic decreased and/or the proportion of Atlantic waters at Site 1006 increased, which may itself be a feedback of increased AMOC strength resulting from restricted Pacific inflow (e.g. refs. 5 and 29). This interpretation for the intervals between 11.5 and 9.5 Ma and between 8 to 6 Ma is supported by independent Mg/Ca based evidence for the presence of a proto-NADW^32^.

**SUPPLEMENTARY REFERENCES**

64. Frank M, Whiteley N, Kasten S, Hein JR, O'Nions K. North Atlantic deep water export to the Southern Ocean over the past 14 Myr: Evidence from Nd and Pb isotopes in ferromanganese crusts. Paleoceanography 17, 1022 (2002).

65. Dausmann V, Frank M, Gutjahr M, Rickli J. Glacial reduction of AMOC strength and long-term transition in weathering inputs into the Southern Ocean since the mid-Miocene: Evidence from radiogenic Nd and Hf isotopes. Paleoceanography 32, 265-283 (2017).
